# A Hot-Melt Extrusion Risk Assessment Classification System for Amorphous Solid Dispersion Formulation Development

**DOI:** 10.3390/pharmaceutics14051044

**Published:** 2022-05-12

**Authors:** Samuel O. Kyeremateng, Kristin Voges, Stefanie Dohrn, Ekaterina Sobich, Ute Lander, Stefan Weber, David Gessner, Rachel C. Evans, Matthias Degenhardt

**Affiliations:** AbbVie Deutschland GmbH & Co. KG, Global Pharmaceutical R&D, Knollstraße, D-67061 Ludwigshafen am Rhein, Germany; kristin.voges@abbvie.com (K.V.); stefanie.dohrn@abbvie.com (S.D.); ekaterina.sobich@abbvie.com (E.S.); ute.lander@abbvie.com (U.L.); stefan.weber@abbvie.com (S.W.); david.gessner@abbvie.com (D.G.); rachel.evans@abbvie.com (R.C.E.); matthias.degenhardt@abbvie.com (M.D.)

**Keywords:** amorphous solid dispersion, hot-melt extrusion, HME risk classification system, HME process design space, quality-by-design, telmisartan, sulfamerazine, copovidone, soluplus, APC™

## Abstract

Several literature publications have described the potential application of active pharmaceutical ingredient (API)–polymer phase diagrams to identify appropriate temperature ranges for processing amorphous solid dispersion (ASD) formulations via the hot-melt extrusion (HME) technique. However, systematic investigations and reliable applications of the phase diagram as a risk assessment tool for HME are non-existent. Accordingly, within AbbVie, an HME risk classification system (HCS) based on API–polymer phase diagrams has been developed as a material-sparing tool for the early risk assessment of especially high melting temperature APIs, which are typically considered unsuitable for HME. The essence of the HCS is to provide an API risk categorization framework for the development of ASDs via the HME process. The proposed classification system is based on the recognition that the manufacture of crystal-free ASD using the HME process fundamentally depends on the ability of the melt temperature to reach the API’s thermodynamic solubility temperature or above. Furthermore, we explored the API–polymer phase diagram as a simple tool for process design space selection pertaining to API or polymer thermal degradation regions and glass transition temperature-related dissolution kinetics limitations. Application of the HCS was demonstrated via HME experiments with two high melting temperature APIs, sulfamerazine and telmisartan, with the polymers Copovidone and Soluplus. Analysis of the resulting ASDs in terms of the residual crystallinity and degradation showed excellent agreement with the preassigned HCS class. Within AbbVie, the HCS concept has been successfully applied to more than 60 different APIs over the last 8 years as a robust validated risk assessment and quality-by-design (Q*b*D) tool for the development of HME ASDs.

## 1. Introduction

The concept of solid dispersions to improve the solubility and bioavailability of poorly water-soluble active pharmaceutical ingredients (APIs) and drugs was first introduced in 1961 by Sekiguchi and Obi [1]. A solid dispersion is typically defined as an intimate dispersion of one or more APIs in an ideally inert carrier, usually a hydrophilic matrix, e.g., a polymer [2]. The high API surface area and improved wettability are substantial contributing factors that lead to the increased dissolution and consequently enhanced bioavailability of solid dispersion formulations. Primarily, there were, and still are, two main methods of preparing solid dispersions, namely melting and solvent-based processes, and extensive reviews have been published [2,3,4,5,6,7,8,9,10].

Fast forward approximately 40 years (early 2000) after the introduction of the solid dispersion formulation concept, the terminology amorphous solid dispersion (ASD) was coined to specifically describe solid dispersions in which the API is amorphously embedded in a polymer matrix [11,12,13]. Besides the increased dissolution described above, ASDs enhance the bioavailability of poorly water-soluble drugs that belong to the biopharmaceutical classification system (BCS) classes II and IV via two effects. First, the amorphous drug exhibits higher apparent solubility compared to its crystalline counterpart. Second, the polymer hinders the thermodynamically non-stable amorphous API against crystallization [14].

Around the same decade, Breitenbach and coworkers started emphasizing the pharmaceutical application of the hot-melt extrusion (HME) technology in the manufacturing of ASDs [15]. Essentially, the HME process involves feeding an API–polymer powder blend into a co-rotating twin-screw extruder. Melting or softening of the polymer matrix occurs due to heating of the barrel housing or viscous dissipation from the shear imparted by the conveying and mixing screw elements. As a result, the API dissolves into the polymer melt through the temperature increase and mixing to form a homogeneous single-phase melt that is extruded through the die and cooled to form an ASD [16,17]. Several published studies have detailed applications of the HME technology in pharmaceutical manufacturing [18,19]. According to recent records, about twelve HME ASD commercial drug products have been approved by the US Food and Drug Administration (FDA) within the last two decades, with about half of them being developed by AbbVie [20]. 

**Figure 1:**
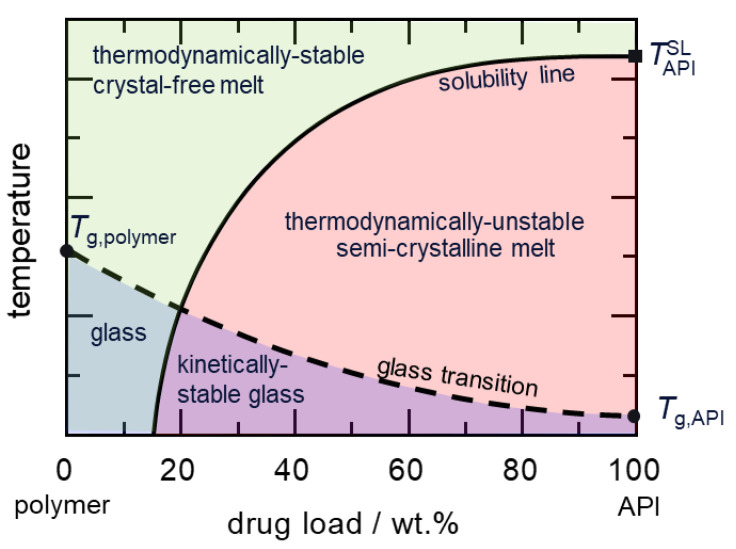
Schematic phase behavior of an API–polymer system. The x-axis represents the DL, “0” means 100% polymer and 0% API, and 100% means 0% polymer and 100% API. The solid curve represents the solubility of the crystalline API in the polymer, while the dashed curve represents the glass transition temperature of the ASD. The curves divide the phase diagram into the following regions: red region: API supersaturated (thermodynamically unstable) semi-crystalline melt; green region: thermodynamically stable crystal-free melt; blue region: thermodynamically stable crystal-free glass; purple region: supersaturated (thermodynamically unstable) kinetically stable glass.

Since the HME process exposes the API and polymer to high temperatures, it is reasonable to assume that very high melting temperatures or thermally labile APIs bear high risk, as often reported in the literature [21,22]. However, it must be emphasized that dissolving an API in a polymer is a thermodynamically driven process. The thermodynamic phase behavior of API–polymer mixtures is well-described in the literature with the Flory–Huggins [23,24] and PC-SAFT [14,25] models. Figure 1 shows a schematic phase diagram of an API–polymer system from 0 to 100% drug load (DL) [14,26].

Above the glass transition (*T*_g_) region in Figure 1, the solubility line delineates the phase diagram into two key regions: the thermodynamically stable crystal-free melt region on the left of the solubility line (green region) and the semi-crystalline or supersaturated melt region on the right of the solubility line (red region). In the latter, the API does not fully dissolve in the polymeric matrix when heating the API–polymer powder blend or can crystallize when cooling from the homogeneous crystal-free melt (green region). Also notable is the glass transition region below the dashed line, where molecular mobility is significantly reduced to the extent that a supersaturated (thermodynamically unstable) system on the right of the solubility line is kinetically stabilized against API crystallization [27]. Conversely, in the blue region, the API is kinetically hindered from dissolving in the polymeric matrix when heating the API–polymer powder blend, although it is on the left of the solubility line and soluble in the polymer from a thermodynamic perspective.

During the HME process, the melt temperature must reach the solubility temperature or above to generate crystal-free ASD [17,28]. Hence, knowledge of the complete API–polymer phase diagram and the API–polymer thermal liability (degradation temperature) is the best approach to a holistic HME risk assessment (HME-RA). Whereas there are several tools and analytical techniques to assess thermal liability and assign risk appropriately, a reliable systematic assessment tool for phase diagrams is missing in the literature. This is partly because the experimental requirements are time-consuming and may take several days to accurately determine solubility temperatures for different API compositions to construct the complete phase diagram [29]. Moreover, in the early phase of pharmaceutical formulation development, the API material may be very limited. Consequently, routine application of the API–polymer phase diagram as a holistic HME-RA tool across the pharmaceutical industry is lacking. 

A few years ago, we developed and reported on a reliable empirical model for generating API–polymer solubility curves based on the API melting temperature and an accurately measured single solubility data point using differential scanning calorimetry (DSC) [30]. This provides a practical and fast option to estimate the solubility curves of a given API in different polymers with minimal experimental efforts, and the model is widely applied in the literature [16,26,29,31,32,33,34,35,36].

Combining the established empirical model with established glass transition models such as the Gordon–Taylor and Fox equations allows the complete generation of API–polymer phase diagrams with minimal API material and experimental effort, thereby enabling the routine generation and application of API–polymer phase diagrams in formulation development. This paper introduces an HME-RA classification system (HCS) based on an API–polymer phase diagram as a novel material-sparing early risk assessment tool for HME ASD formulation development. Furthermore, the application of the API–polymer phase diagram in identifying the HME process design space in relation to two key HME ASD formulation critical quality attributes (CQAs), namely the residual crystallinity and chemical–thermal stability, is demonstrated with high melting temperature (>200 °C) model APIs.

## 2. Materials and Methods

### 2.1. Materials

Telmisartan was purchased from Molekula Limited (Darlington, UK) and sulfamerazine from Sigma-Aldrich (Steinheim, Germany). HPLC-grade tetrahydrofuran (THF) was purchased from Sigma-Aldrich (Steinheim, Germany). Copovidone and Soluplus were purchased from BASF (Ludwigshafen, Germany).

### 2.2. Preparation of API–Polymer Physical Mixtures for Thermal Analysis 

The cryomill Freezer/Mill 6750 (SPEX SamplePrep, Metuchen, NJ, USA) was used for preparing API–polymer physical mixtures for thermal analysis. The API and polymer with a total mass of 200 mg were weighed into microvials for cryomilling. The samples were pre-cooled for 5 min and then milled at 10 Hz for 10 min in five cycles. For sulfamerazine, physical mixtures containing 40 wt.% of the API in either Copovidone or Soluplus were prepared. With telmisartan, physical mixtures containing 15 wt.% and 5 wt.% of the API in Copovidone and Soluplus, respectively, were prepared.

### 2.3. Differential Scanning Calorimetry (DSC)

A Mettler-Toledo DSC 1 instrument (Mettler-Toledo, GmbH, Giessen, Germany) with a Huber TC100 (Huber Kältemaschinenbau AG, Offenburg, Germany) immersion cooler and auto-sampler was used for thermal analysis of the API, polymer, and API–polymer physical mixtures. Nitrogen at a flow rate of 50 mL min^−1^ was used as a purging gas, and 40 µL aluminum pans with pierced lids were used for the samples. To measure the melting temperature and the *T*_g_ of pure API, 3–5 mg of the API was heated at 10 K min^−1^ from 25 °C to 250 °C and 280 °C for sulfamerazine and telmisartan, respectively. This was followed by a fast cooling stage from 50 K min^−1^ to −60 °C and then reheating at 10 K min^−1^. The melting temperature of the API is reported as the peak temperature of the endothermic melting signal during the first heating. The *T*_g_ of the amorphous API was determined from the half-height of the transition step in the thermogram from the second heating. To determine the solubility of the APIs in the polymers, 10–15 mg of the cryomilled physical mixture was heated from 25 °C to 280 °C at a heating rate of 1.5 K min^−1^. Due to the particle size reduction and homogenization via cryomilling, coupled with the slow heating rate, kinetic limitations on the API dissolution process were minimized. Thus, the end-set of the endothermic dissolution signal was considered the API’s solubility temperature. To measure the *T*_g_ of the ASD, 10–15 mg of the cryomilled physical mixture was heated at 10 K min^−1^ from 25 °C to 10 °C above the respective solubility temperature. The sample was then cooled to −60 °C at a cooling rate of −50 K min^−1^ and reheated. The glass transition temperature of the ASD was determined from the half-height of the transition step in the thermogram from the second heating phase.

### 2.4. Thermogravimetric Analysis (TGA)

Thermal degradation of the APIs and polymers was measured using a TGA/DSC1 instrument (Mettler-Toledo, GmbH, Giessen, Germany) at a heating rate of 10 K min^−1^ from 25 °C to 300 °C under nitrogen gas purging (50 mL min^−1^) and with sample weights of 10–20 mg.

### 2.5. HME

**Figure 2:**
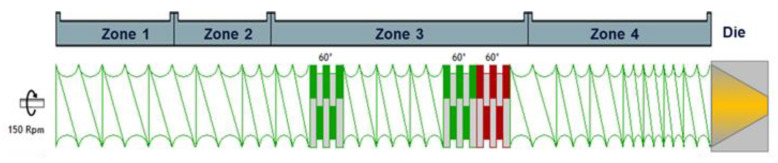
Extruder geometry and screw configuration. Note: Die and screw depictions are not to scale. Drawings prepared with Ludovic^®^ v.6.0 software (SC-Consultants, Saint-Etienne, France). Green kneading blocks are 60° forward, while red kneading blocks are 60° backward.

For each formulation, the extrusion blend components (API and polymer) were individually weighed and sieved through a 500 µm screen to ensure homogeneity and particle size similarity. Subsequently, the components in their respective weight ratios with a total mass of 250 g were weighed in a 1 L glass vessel and blended with an Inversina Tumbler Mixer (Bioengineering AG, Wald, Switzerland) at 10 rpm for 2 min. The blend was then hand-sieved through an 800 µm screen to remove agglomerates and to ensure homogeneity, before being blended for another 2 min with the Inversina Tumbler Mixer at 15 rpm. The Rondol 10 mm microextruder (Rondol Industrie, Strasbourg, France) was used for extrusion at a screw speed of 150 rpm and feed rate of 100 g h^−1^ with a batch size of approximately 250 g. The extruder geometry and screw configuration are schematically shown in Figure 2.

The barrel of the extruder contained four temperature zones and a die: zone 1 was set to 80 °C, while zones 2–4 and the die were set to 140 °C, 160 °C, 180 °C, 200 °C, 220 °C, or 230 °C across.

### 2.6. Polarized Light Microscopy (PLM)

Extrudate splinters were obtained by grinding the extrudates in an agate mortar. The splinters were spread onto a glass slide, and PLM images were taken with a Leica DMLM microscope (Leica Mikrosysteme Vertrieb GmbH, Wetzlar, Germany) equipped with N Plan 10x/0.25 objective, transmitted light, polarizers, and a Leica DFC 320 camera.

### 2.7. X-Ray Powder Diffraction (XRPD)

An Empyrean X-ray powder diffractometer from Malvern Panalytical, fitted with a copper tube and a Pixel 3D Medipix3 detector, was used to measure the crystallinity of the extrudates in reflection geometry mode. The extrudates were briefly cryomilled and placed in the XRPD backloading sample holder. The measurements were performed in Bragg Brentano geometry with Cu Kα radiation (45 KV × 40 mA). The diffraction pattern was recorded in the range of 5–25°2θ with a step size of 0.026°2θ and a measurement time of 4000 s per step. 

### 2.8. Advanced Polymer Chromatography™ (APC™)

The APC™ system from Waters Corporation (Milford, MA, USA) was employed to simultaneously monitor the thermal stability of the polymers and APIs post-extrusion. The ACQUITY PDA detector (190–400 nm, resolution 1.2 nm) of the system was used to monitor the elution of the polymers, sulfamerazine, and telmisartan at wavelengths of 210 nm, 250 nm, and 295 nm, respectively, using THF as the mobile phase at a flow rate of 0.9 mL min^−1^. The column setup in the system consisted of a 0.2 µL pre-column filter, one ACQUITY APC™ XT 200 Å 2.5 µm 4.6 mm × 150 mm column, and two ACQUITY APC™ XT 45 Å 2.5 µm 4.6 mm × 150 mm columns. The sample concentration was 1 mg/mL and 10 µL sample volume was injected. Measurements were performed at 40 °C in triplicate. A 5-point calibration curve for the API was generated for each formulation composition. Data acquisition and analysis were performed using Empower 3 (Waters Corp., Milford, MA, USA).

## 3. Modeling

### 3.1. Modeling API Solubility in Polymer

The presented empirical approach for calculating the solubility of an API in a polymer was developed by Kyeremateng et al. [30]:

(1) $$T^{\mathrm{SL}}=-A\;e^{-0.05w_{API}}+T_{o,\mathrm{API}}^{\mathrm{SL}}+C$$

where $T_{o,\mathrm{API}}^{\mathrm{SL}}$ is the melting temperature of the API; *T*^SL^ is the solubility temperature for a specific API load *w*_API_ (wt. %) in the polymer; *A* and *C* are fitting parameters [26,30], which are fitted to experimental data. Table 1 lists the measured melting temperature, *T*_g_ (Section 2.3), and thermal degradation temperature (Section 2.4) of the studied APIs and polymers. Table 2 lists the measured solubility temperature, ASD *T*_g_, and fitting parameters from Equation (1) of the API–polymer systems.

### 3.2. Modeling Glass Transition Temperature

The glass transition of API–polymer ASDs (*T*_g, mix_) was predicted using the Fox Equation (Equation (2))) [37]:

(2) $$1/T_{g,\mathrm{mix}}=\underset{i}{\sum }w_{i}/T_{g,i}$$

## 4. Results and Discussions

**Figure 3:**
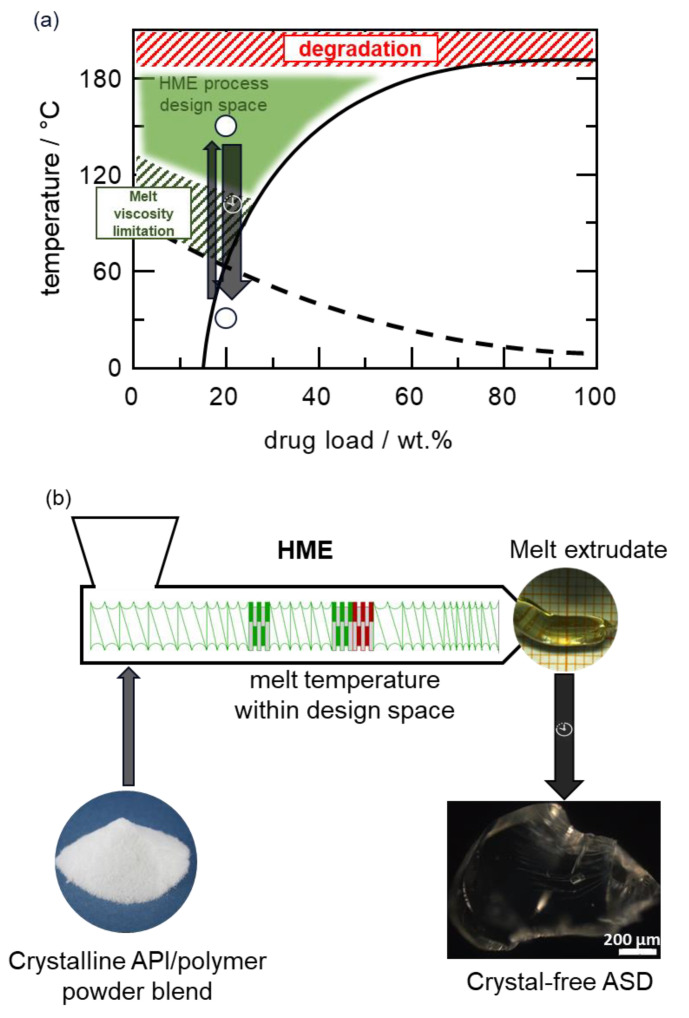
Schematic API–polymer phase diagram in relation to the HME process design space. (**a**) API–polymer phase diagram with the solid curve representing the API solubility in the polymer and the dashed curve the glass transition temperature. The red dashed region defines the upper boundary temperature limit of the HME process due to thermal degradation; the green dashed region defines the lower boundary of the HME process due to the high viscosity of the melt at temperatures close to the glass transition temperature. The green region above the solubility line represents the HME process design space. The two open circles represent the HME process pathway in relation to the phase diagram for an exemplary ASD with a 20 wt.% DL. The API–polymer powder blend fed into the extruder must be processed at melt temperature within the design space and cooled back to ambient temperature as schematically depicted (**b**) to manufacture a crystal-free ASD.

The two essential critical quality attributes (CAQs) of ASD drug products that must be fulfilled for a successful HME process are avoiding residual crystalline API in the melt and thermal degradation. Figure 3 schematically illustrates how the API–polymer phase diagram can be applied in defining the HME process design space to meet these CQAs.

Initially, the API–polymer powder blend mixture is fed into the extruder via the hopper at ambient conditions. Within the extruder, the mixture must be processed at temperatures within the defined green region to obtain a crystal-free melt without degradation, as exemplified for a 20 wt% drug load (DL) (Figure 3a). It must be emphasized that the API can practically dissolve into the polymer matrix when the polymer transitions into the melt phase well above its *T*_g_, which defines the lower boundary of the design space. Within this lower boundary, the API dissolution kinetics is significantly slow due to the high melt viscosity, such that the dissolution process cannot be completed within the residence time scale of the extrusion process. The upper limit of the process design space is bound by the thermal degradation temperature, as depicted in Figure 3a. Figure 3b schematically illustrates the relation between the design space and the HME process. 

**Figure 4:**
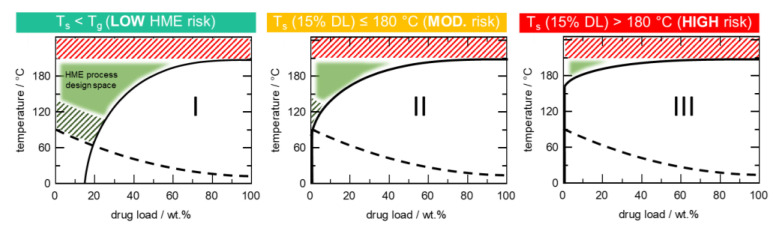
Schematic HME risk classification system (HCS) based on API–polymer phase diagrams. The solid curve represents the API’s solubility in the polymer, while the dashed curve represents the *T*_g_ of the ASD. Class I: Low HME risk with significant API solubility below polymer’s *T*_g_. Class II: Moderate HME risk since 15 wt.% DL solubility temperature is ≤180 °C. Class III: High HME risk since 15 wt.% DL solubility temperature is >180 °C.

To provide a streamlined early risk assessment of APIs, especially high melting temperature (>180 °C) APIs being considered for the HME process, we have developed a novel classification scheme based on API–polymer phase diagrams. Figure 4 introduces the HME risk classification system (HCS), which is categorized into three classes, namely class I, class II, and class III.

The proposed HCS is based on the recognition that the successful manufacturing of crystal-free ASD by HME fundamentally depends on the ability to reach a melt temperature close to or above the solubility temperature of the API in the excipient matrix. From a thermodynamic perspective, residual API crystals will be present if the melt temperature is lower than the solubility temperature. The implementation of HCS in the development of ASDs offers an advantage in conserving resources and minimizing the number of experimental trials required to produce ASD drug products that meet CQAs.

Most commercial ASD oral drug products contain 10 to 20 wt.% DL relative to the polymer [38,39,40,41,42], and most excipients used for ASD formulations, besides Copovidone and Soluplus, tend to be thermally unstable beyond 180 °C [19,43]. Based on that premise, 15 wt.% DL and 180 °C were adopted as reference baselines for the HCS. 

HCS class I refers to an API with significant solubility below the *T*_g_ of the polymer. Such an API is described as having high solubility in the polymer. Class I systems are generally classified as low risk for HME. They are less likely to encounter extrusion challenges in generating crystal-free extrudate at melt temperatures well below 180 °C for 15 wt.% DL. Technically, this class also includes APIs with melting temperatures well below 180 °C, such as ibuprofen, fenofibrate, and naproxen, to mention a few [30]. 

HCS class II describes systems where the API has insignificant solubility below *T*_g_ of the polymer but shows a significant solubility close to and above *T*_g_ of the polymer with the solubility temperature for 15 wt.% DL ≤180 °C. HCS class II systems are, therefore, described as having moderate HME risk in generating crystal-free extrudate. 

HCS class III refers to systems with the API showing low solubility in the polymer. For such systems, significant API solubility in the polymer is only achievable at temperatures far above the *T*_g_ of the polymer and usually close to the melting temperature of the API. Hence, HCS class III systems have a high HME risk in generating crystal-free extrudate, as the solubility temperature for 15 wt.% DL is >180 °C.

### 4.1. HCS Class I System

**Figure 5:**
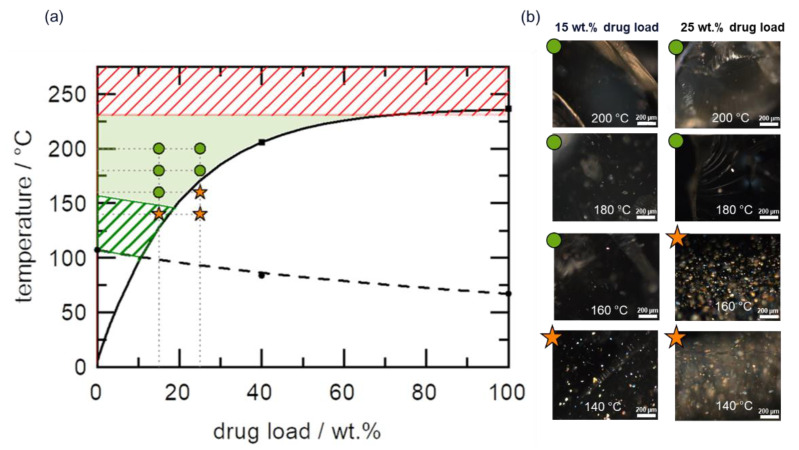
(**a**) Sulfamerazine–Copovidone phase diagram. The solid curve represents the solubility line calculated using the empirical model proposed by Kyeremateng et al. [30]. The dashed curve represents the glass transition temperature calculated using the Fox equation. The black symbols are experimental data points of the solubility temperature (squares) and glass transition temperature (circles) used for the modeling. The green region indicates the HME process design space, while the green and red shaded regions represent the lower and upper boundaries of the design space, respectively. The green circle and orange star symbols reflect PLM amorphous and crystalline extrudates, respectively (**b**), after HME of 15 wt.% and 25 wt.% DL formulations at 140 °C, 160 °C, 180 °C, and 200 °C.

Due to the high melting temperature of sulfamerazine (236 °C), coupled with the fact that Copovidone thermally degrades above 230 °C, it is reasonable to assume that HME is not a suitable technology for sulfamerazine–Copovidone ASD manufacturing. However, Figure 5a shows that the sulfamerazine–Copovidone phase diagram belongs to HCS class I, thus, showing significant API solubility below *T*_g_ of the polymer resulting in low HME risk. 

The predicted HME process design space (green region in Figure 5a) is bound on the upper limit by the thermal stability limit of Copovidone at 230 °C (sulfamerazine thermally degrades at 246 °C). As previously mentioned, the lower limit of the design space in this case is bound by the limiting slow API dissolution kinetics due to the high viscosity of the polymer melt in the region just above *T*_g_. The dissolution kinetics typically improves around *T*_g_ + 50 °C. 

HME experiments were conducted with 15 wt.% and 25 wt.% sulfamerazine in Copovidone for conditions within and outside the design space to verify the process design space. As seen in the PLM images in Figure 5b (full PLM images are provided in Figure S1), the extrudate samples processed at temperatures outside the design space showed birefringence due to residual API crystals. In contrast, samples processed within the design space were completely amorphous. Examination of the PLM image of the 15 wt.% sample extruded at 140 °C indicated relatively fewer residual API crystals, while the XRPD diffractogram (Figure S2) revealed only an amorphous halo because XRPD is comparatively less sensitive to residual API crystals than PLM [25]. The observed minimal residual crystals in the 15 wt.% formulation processed at 140 °C agree with the fact that the API is thermodynamically soluble in the polymer (above solubility curve) at this temperature. However, the anticipated slow API dissolution kinetics in the lower boundary of the design space leads to incomplete API dissolution. APC™ results confirmed that API degradation did not occur, as the API assays of all extrudates were in the range of 98–100%. Overall, the HME process design space predicted for this HCS class I system agrees well with the extrusion results.

### 4.2. HCS Class II System

**Figure 6:**
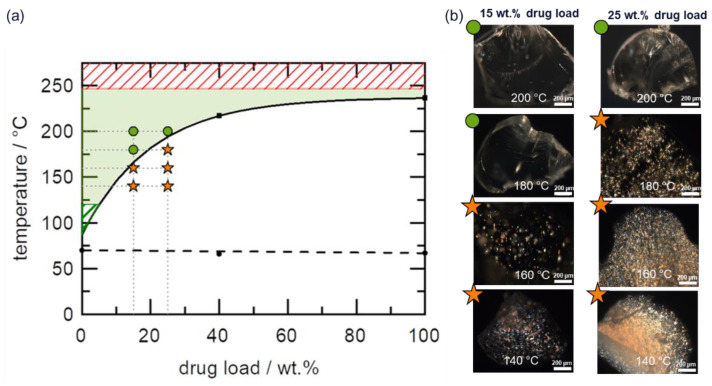
(**a**) Sulfamerazine–Soluplus phase diagram. The solid curve represents the solubility line calculated using the empirical model proposed by Kyeremateng et al. [30]. The dashed curve represents the glass transition temperature calculated using the Fox equation. The black symbols are the experimental data for the solubility temperature (squares) and glass transition temperature (circles) used for the modeling. The green region indicates the HME process design space, while the green and red shaded regions represent the lower and upper boundaries of the design space, respectively. The green circle and orange star symbols reflect PLM amorphous and crystalline extrudates, respectively (**b**), after HME of 15 wt.% and 25 wt.% DL formulations at 140 °C, 160 °C, 180 °C, and 200 °C.

Paring the polymer Soluplus with sulfamerazine leads to an HCS class II phase diagram, as shown in Figure 6a. The API has insignificant solubility below the *T*_g_ of the polymer but shows significant solubility above the polymer’s *T*_g_. 

The system has moderate HME risk because the solubility temperature readout from the phase diagram for 15 wt.% DL is less than the HCS high risk defined threshold temperature of >180 °C. Specifically, the solubility temperature of 15 wt.% sulfamerazine in Soluplus is 166 °C, which is higher than the corresponding Copovidone-based HCS I system (Figure 5a).

The predicted HME design space is bound narrowly on the lower limit by the anticipated slow API dissolution kinetics at temperatures close to *T*_g_, while the upper limit is defined by the thermal degradation temperature of sulfamerazine of 246 °C (Soluplus thermally degrades at 250 °C). The HME of 15 wt.% and 25 wt.% DL formulations was performed to verify the design space, and the results are shown in Figure 6b. As expected, extruded formulations below the solubility temperature and outside the design space exhibited residual API crystals in the PLM images and were confirmed by XRPD (Figure S2). Conversely, all extruded formulations within the design space appeared completely amorphous in the PLM images. Additionally, APC™ results confirmed that the API assays of the extrudates were in the range of 98–100%, indicating that API degradation did not occur during HME. In essence, the extrusion results indeed confirmed the predicted HME process design space.

### 4.3. HCS Class III System

**Figure 7:**
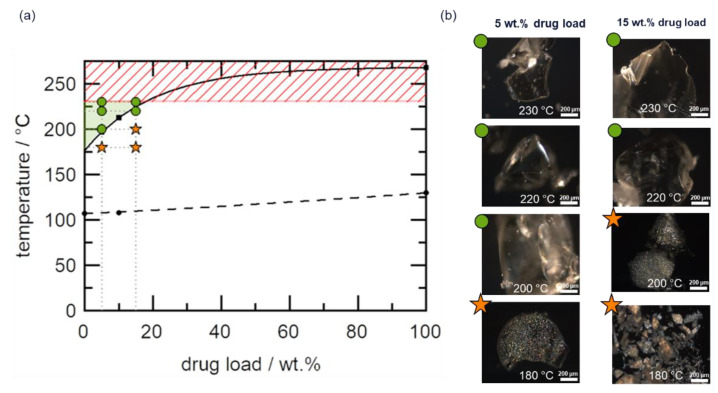
(**a**) Telmisartan–Copovidone phase diagram. The solid curve represents the solubility line calculated using the empirical model proposed by Kyeremateng et al. [30]. The dashed curve represents the glass transition temperature calculated using the Fox equation. The black symbols are the experimental data for the solubility temperature (squares) and glass transition temperature (circles) used for the modeling. The green region indicates the process design space, while the red shaded region represents the upper boundary of the design space. The green circle and orange star symbols reflect PLM amorphous and crystalline extrudates, respectively (**b**), after HME of 5 wt.% and 15 wt.% DL formulations at 180 °C, 200 °C, 220 °C, and 230 °C.

Figure 7a shows the phase diagram of the telmisartan–Copovidone system, which is an HCS class III system, because the API solubility in the polymer is only achievable at temperatures well-above the *T*_g_ of the polymer, whereby the solubility temperature for 15 wt.% DL is >180 °C, indicating high HME risk. A narrow HME process design space was identified, which is bound on the lower and upper limits by the minimum solubility temperature (175 °C for a DL close to 0 wt%) and Copovidone’s thermal degradation temperature (230 °C), respectively, as depicted in Figure 7a. It is wort noting that the lower boundary of the design space in this case is defined by thermodynamics (minimum solubility temperature) rather than API dissolution kinetics limitations at temperatures close to *T*_g_, which was the case for the HCS class I system and to a minimal extent the HCS class II system.

**Figure 8:**
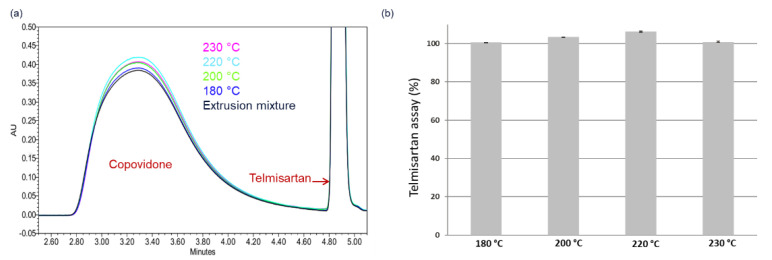
(**a**) APC™ overlay chromatograms of 15wt.% telmisartan–Copovidone pre-extrusion blend (black) and extrudates processed at 180 °C (blue), 200 °C (green), 220 °C (light blue), and 230 °C (pink). (**b**) Telmisartan assay plot for extrudates processed at 180 °C, 200 °C, 220 °C, and 230 °C.

The validity of the narrow process design space was explored by HME of 5 wt.% and 15 wt.% DL formulations. As shown in Figure 7b, residual API crystals were detected in PLM images of all extrudates processed at temperatures outside the design space (Figure 7a) and confirmed by XRPD (Figure S4). As expected, all extrudates processed within the design space appeared completely amorphous, as evidenced by the lack of birefringence in the corresponding PLM images shown in Figure 7b (full PLM images are provided in Figure S3). Since the extrudates were manufactured at temperatures close to the upper boundary of the design space, it is imperative in this case to assess the thermal stability of both polymer and API post-extrusion. Figure 8a shows the APC™ chromatogram overlays of the pre-extrusion blend of the 15 wt.% DL formulation and the corresponding extrudates manufactured at 180 °C to 230 °C, while the resulting API assays are shown in Figure 8b.

The APC™ chromatograms (Figure 8a) show the broad peak corresponding to Copovidone, which maintained its peak position and shape, suggesting that the polymer remained stable under all extrusion conditions, indicating no significant polymer degradation. Likewise, the sulfamerazine assay remained on target (~100%) for all extrusion conditions, as shown in Figure 8b, equally indicating no API degradation. Thus, the narrowly defined process design predicted for this high-risk HCS class III system was validated.

**Figure 9:**
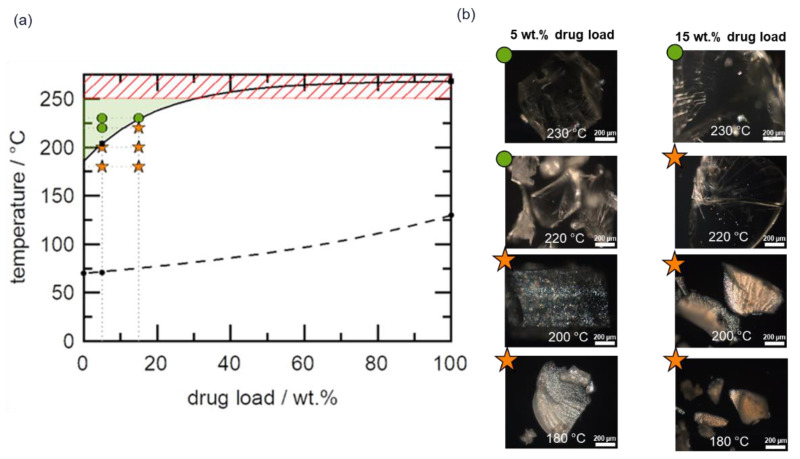
(**a**) Telmisartan–Soluplus phase diagram. The solid line represents the solubility line calculated using the empirical model proposed by Kyeremateng et al. [30]. The dashed curve represents the glass transition temperature calculated using the Fox equation. The black symbols are the experimental data for the solubility temperature (squares) and glass transition temperature (circles) used for the modeling. The green region indicates the process design space, while the red shaded region represents the upper boundary of the design space. The green circle and orange star symbols reflect PLM amorphous and crystalline extrudates, respectively (**b**), after HME of 5 wt.% and 15 wt.% DL formulations at 180 °C, 200 °C, 220 °C, and 230 °C.

Switching the polymer to Soluplus still led to a high-risk HCS class III system for telmisartan, as shown in Figure 9a. Similarly, a narrow HME process design space was identified, which is bound on the lower and upper limits by the minimum solubility temperature (186 °C) and Soluplus’ thermal degradation temperature (250 °C), respectively, as depicted in Figure 9a. Compared with the minimum solubility temperature of the telmisartan–Copovidone system in Figure 7a, it appears telmisartan is less soluble in Soluplus than in Copovidone.

**Figure 10:**
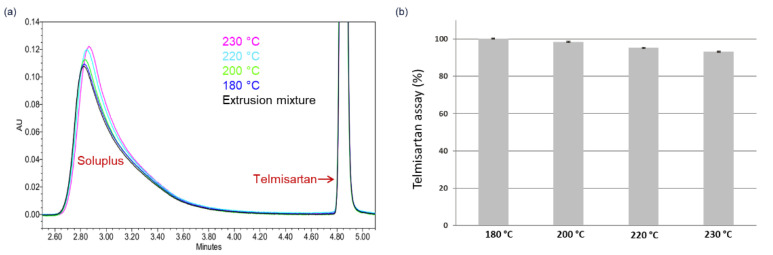
(**a**) APC™ overlay chromatograms of 15 wt.% telmisartan–Soluplus pre-extrusion blend (black) and extrudates processed at 180 °C (blue), 200 °C (green), 220 °C (light blue), and 230 °C (pink). (**b**) Telmisartan assay plot for extrudates processed at 180 °C, 200 °C, 220 °C, and 230 °C.

Similarly, the validity of the process design space was explored by HME of 5 wt.% and 15 wt.% DL formulations. Residual API crystals were detected in PLM images of all extrudates processed at temperatures outside the design space, as seen in Figure 9b and confirmed by XRPD results in Figure S4. All extrudates processed within the design space appeared completely amorphous, as seen in the PLM images in Figure 9b (full PLM images are provided in Figure S3). Like the Copovidone system, the thermal stability of Soluplus and the API post-extrusion were assessed. Figure 10a shows the APC™ chromatogram overlays of the pre-extrusion blend of the 15 wt.% DL formulation and the corresponding extrudates manufactured at 180 °C to 230 °C. The broad peak corresponding to Soluplus in the chromatograms shifted to a higher retention time for the extrudates manufactured at 220 °C and above, suggesting degradation of the polymer. Likewise, significant telmisartan assay loss was observed, as seen in Figure 10b, leading to 95% and 93% assays for the extrudates processed at 220 °C and 230 °C, respectively.

It is apparently clear that Soluplus induced telmisartan’s degradation, since this was not the case when telmisartan was extruded with Copovidone above 200 °C. Thus, characterization of the ASD with chromatographic techniques such as APC™ is required to identify the synergistic degradation of API and polymer below their respective degradation temperatures, especially for high-risk HCS class III systems.

## 5. Conclusions

The rationale for the selection of hot-melt extrusion (HME) technology for manufacturing amorphous solid dispersion (ASD) formulations is commonly based on the API’s melting temperature. As a result, the HME process is often ruled out for many APIs with high melting temperatures due to the risk of thermally driven degradation of the API and polymer at high temperatures. This ignores the fact that depending on the specific API–polymer phase diagram, the thermodynamic temperature required to amorphously embed the API in the polymer can be significantly lower than the API’s melting temperature. The introduced simple yet powerful HME risk classification system (HCS) provides a framework for the early risk assessment of ASD manufacturability by HME as opposed to API’s melting temperature. Knowledge gained from the HCS can be applied using the quality-by-design (Q*b*D) approach to identify and select the HME process design space to meet two key critical quality attributes (CQAs) of the ASD formulation—crystallinity and degradation. The application of the HCS was successfully demonstrated with two high melting point APIs, telmisartan and sulfamerazine, using two common HME polymers, Copovidone and Soluplus. It was shown that residual API crystals and thermal degradation can be avoided during ASD manufacturing of these challenging APIs if the predicted HME process design space is maintained. Nonetheless, characterization of the formulations with chromatographic techniques such as the APC™ is required to identify the potential synergistic degradation of API and polymer below their respective degradation temperatures. The concepts presented here were applied to other high melting point APIs, such as pibrentasvir, in the development of HME ASD by AbbVie.

## Figures and Tables

**Table 1:** Melting temperature, degradation temperature, and *T*_g_ values of APIs and polymers investigated in this work.

	Melting Temperature/°C	Thermal Degradation Temperature/°C	*T*_g_/°C
Telmisartan	269	280	129
Sulfamerazine	236	246	129
Copovidone	-	230	107
Soluplus	-	250	70

**Table 2:** DSC-measured solubility temperatures of the API–polymer mixtures and *T*_g_ values of corresponding ASDs. Dimensionless A and C parameters applied in Equation (1) for the solubility curve fitting.

API–Polymer System	Drug Load (DL)/wt.%	Solubility Temperature ^1^/°C	*T*_g_/°C	Parameter *A*	Parameter *C*
Sulfamerazine–Copovidone	40	206.0	83.8	231.7	1
Sulfamerazine–Soluplus	40	217.0	66.0	150.4	1
Telmisartan–Copovidone	10	212.7	107.8	91.2	0
Telmisartan–Soluplus	5	203.7	70.7	82.5	0

^1^ APC analysis confirmed no API degradation at solubility temperature.

## Supplementary Materials

The following supporting information can be downloaded at: https://www.mdpi.com/article/10.3390/pharmaceutics14051044/s1: Figure S1: Polarized light microscopy (PLM) images of extrudates; 15 wt.% and 25 wt. % sulfamerazine (a) in Copovidone and (b) in Soluplus. The green circle and orange star symbols reflect PLM amorphous and crystalline extrudates, respectively, after HME at 180 °C, 200 °C, 220 °C, and 230 °C. Figure S2: XRPD data for extrudates: 15 wt.% sulfamerazine (a) in Copovidone and (b) in Soluplus and 25 wt.% sulfamerazine in (c) Copovidone and (d) Soluplus. Figure S3: Polarized light microscopy (PLM) images of extrudates; 5 wt.% and 15 wt. % telmisartan (a) in Copovidone and (b) in Soluplus. The green circle and orange star symbols reflect PLM amorphous and crystalline extrudates, respectively, after HME at 180 °C, 200 °C, 220 °C, and 230 °C. Figure S4: XRPD data for extrudates: 5 wt.% telmisartan (a) in Copovidone and (b) in Soluplus and 15 wt.% telmisartan in (c) Copovidone and (d) Soluplus.

## Abbreviations

*A*	Dimensionless fitting parameter
APC™	Advanced Polymer Chromatography™
API	Active pharmaceutical ingredient
ASD	Amorphous solid dispersion
*C*	Dimensionless fitting parameter
CQA	Critical quality attribute
DL	Drug load
HCS	HME risk classification system
HME	Hot-melt extrusion
*K*	Kelvin
PLM	Polarized light microscopy
Q*b*D	Quality-by-design
$T^{\mathrm{SL}}$	Solubility temperature
*T*_g_	Glass transition temperature
$T_{o,\mathrm{API}}^{\mathrm{SL}}$	API melting temperature
*w*_API_	API load
XRPD	X-ray powder diffraction

## References

[1] Sekiguchi K., Obi N. (1961). Studies on Absorption of Eutectic Mixture. I. A Comparison of the Behavior of Eutectic Mixture of Sulfathiazole and that of Ordinary Sulfathiazole in Man. Chem. Pharm. Bull..

[2] Ford J.L. (1986). The current status of solid dispersions. Pharma. Acta Helv..

[3] Alam M.A., Ali R., Al-Jenoobi F.I., Al-Mohizea A.M. (2012). Solid dispersions: A strategy for poorly aqueous soluble drugs and technology updates. Expert Opin. Drug Deliv..

[4] Allawadi D., Singh N., Singh S., Arora S. (2013). Solid dispersions: A review on drug delivery system and solubility enhancement. IJPSR.

[5] Mendonsa N., Almutairy B., Kallakunta V.R., Sarabu S., Thipsay P., Bandari S., Repka M.A. (2020). Manufacturing strategies to develop amorphous solid dispersions: An overview. J. Drug Deliv. Sci. Technol..

[6] Schittny A., Huwyler J., Puchkow M. (2020). Mechanisms of increased bioavailability through amorphous solid dispersions: A review. Drug Deliv..

[7] Tran P., Pyo Y.-C., Kim D.-H., Lee S.-E., Kim J.-K., Park J.-S. (2019). Overview of the Manufacturing Methods of Solid Dispersion Technology for Improving the Solubility of Poorly Water-Soluble Drugs and Application to Anticancer Drugs. Pharmaceutics.

[8] Vasconcelos T., Marques S., das Neves J., Sarmento B. (2016). Amorphous solid dispersions: Rational selection of a manufacturing process. Adv. Drug Deliv. Rev..

[9] Vo C.L.-N., Park C., Lee B.-J. (2013). Current trends and future perspectives of solid dispersions containing poorly water-soluble drugs. Eur. J. Pharm. Biopharm..

[10] Zhang J., Han R., Chen W., Zhang W., Ji Y., Chen L., Pan H., Yang X., Pan W., Ouyang D. (2018). Analysis of the Literature and Patents on Solid Dispersions from 1980 to 2015. Molecules.

[11] Ingkatawornwong S., Kaewnopparat N., Tantishaiyakul V. (2001). Studies on aging piroxicam-polyvinylpyrrolidone solid dispersions. Die Pharm..

[12] Appel L., Curatolo W.J., Herbig S.M., Nightingale J., Thombre A. (2000). Osmotic System for Delivery of Solid Amorphous Dispersions of Drugs. European Patent.

[13] Tantishaiyakul V., Kaewnopparat N., Ingkatawornwong S. (1999). Properties of solid dispersions of piroxicam in polyvinylpyrrolidone. Int. J. Pharm..

[14] Prudic A., Ji Y., Sadowski G. (2014). Thermodynamic Phase Behavior of API/Polymer Solid Dispersions. Mol. Pharm..

[15] Breitenbach J. (2002). Melt extrusion: From process to drug delivery technology. Eur. J. Pharm. Biopharm..

[16] Evans R.C., Bochmann E.S., Kyeremateng S.O., Gryczke A., Wagner K.G. (2019). Holistic QbD approach for hot-melt extrusion process design space evaluation: Linking materials science, experimentation and process modeling. Eur. J. Pharm. Biopharm..

[17] Evans R.C., Kyeremateng S.O., Asmus L., Degenhardt M., Rosenberg J., Wagner K.G. (2018). Development and Performance of a Highly Sensitive Model Formulation Based on Torasemide to Enhance Hot-Melt Extrusion Process Understanding and Process Development. AAPS PharmSciTech.

[18] Maniruzzaman M., Boateng J.S., Snowden M.J., Douroumis D. (2012). A review of hot-melt extrusion: Process technology to pharmaceutical products. ISRN Pharm..

[19] Kolter K., Karl M., Gryczke A. (2012). Hot-Melt Extrusion with BASF Pharma Polymers.

[20] Bhujbal S.V., Mitra B., Jain U., Gong Y., Agrawal A., Karki S., Taylor L.S., Kumar S., Tony Zhou Q. (2021). Pharmaceutical amorphous solid dispersion: A review of manufacturing strategies. Acta Pharm. Sinica. B.

[21] Thompson S.A., Davis D.A., Moon C., Williams R.O. (2022). Increasing Drug Loading of Weakly Acidic Telmisartan in Amorphous Solid Dispersions through pH Modification during Hot-Melt Extrusion. Mol. Pharm..

[22] Tiwari R.V., Patil H., Repka M.A. (2016). Contribution of hot-melt extrusion technology to advance drug delivery in the 21st century. Expert Opin. Drug Deliv..

[23] Lin D., Huang Y. (2010). A thermal analysis method to predict the complete phase diagram of drug-polymer solid dispersions. Int. J. Pharm..

[24] Tian Y., Booth J., Meehan E., Jones D.S., Li S., Andrews G.P. (2013). Construction of drug-polymer thermodynamic phase diagrams using Flory-Huggins interaction theory: Identifying the relevance of temperature and drug weight fraction to phase separation within solid dispersions. Mol. Pharm..

[25] Lehmkemper K., Kyeremateng S.O., Heinzerling O., Degenhardt M., Sadowski G. (2017). Impact of Polymer Type and Relative Humidity on the Long-Term Physical Stability of Amorphous Solid Dispersions. Mol. Pharm..

[26] Lehmkemper K., Kyeremateng S.O., Heinzerling O., Degenhardt M., Sadowski G. (2017). Long-Term Physical Stability of PVP- and PVPVA-Amorphous Solid Dispersions. Mol. Pharm..

[27] Hancock B.C., Shamblin S.L., Zografi G. (1995). Molecular Mobility of Amorphous Pharmaceutical Solids Below Their Glass Transition Temperatures. Pharm. Res..

[28] Moseson D.E., Taylor L.S. (2018). The application of temperature-composition phase diagrams for hot melt extrusion processing of amorphous solid dispersions to prevent residual crystallinity. Int. J. Pharm..

[29] Thakore S.D., Akhtar J., Jain R., Paudel A., Bansal A.K. (2021). Analytical and Computational Methods for the Determination of Drug-Polymer Solubility and Miscibility. Mol. Pharm..

[30] Kyeremateng S.O., Pudlas M., Woehrle G.H. (2014). A fast and reliable empirical approach for estimating solubility of crystalline drugs in polymers for hot melt extrusion formulations. J. Pharm. Sci..

[31] Iemtsev A., Hassouna F., Mathers A., Klajmon M., Dendisová M., Malinová L., Školáková T., Fulem M. (2020). Physical stability of hydroxypropyl methylcellulose-based amorphous solid dispersions: Experimental and computational study. Int. J. Pharm..

[32] Theil F., Anantharaman S., Kyeremateng S.O., van Lishaut H., Dreis-Kühne S.H., Rosenberg J., Mägerlein M., Woehrle G.H. (2017). Frozen in Time: Kinetically Stabilized Amorphous Solid Dispersions of Nifedipine Stable after a Quarter Century of Storage. Mol. Pharm..

[33] Ojo A.T., Lee P.I. (2021). A Mechanistic Model for Predicting the Physical Stability of Amorphous Solid Dispersions. J. Pharm. Sci..

[34] Mathers A., Hassouna F., Klajmon M., Fulem M. (2021). Comparative Study of DSC-Based Protocols for API-Polymer Solubility Determination. Mol. Pharm..

[35] Mathers A., Hassouna F., Malinová L., Merna J., Růžička K., Fulem M. (2020). Impact of Hot-Melt Extrusion Processing Conditions on Physicochemical Properties of Amorphous Solid Dispersions Containing Thermally Labile Acrylic Copolymer. J. Pharm. Sci..

[36] Ma X., Huang S., Lowinger M.B., Liu X., Lu X., Su Y., Williams R.O. (2019). Influence of mechanical and thermal energy on nifedipine amorphous solid dispersions prepared by hot melt extrusion: Preparation and physical stability. Int. J. Pharm..

[37] Fox T.G. (1956). Influence of Diluent and of Copolymer Composition on the Glass Temperature of a Polymer System. Bull. Am. Phys. Soc..

[38] Britalan E., Hoelig P., Lindley D.J., Sanzgiri Y.D., Tong P. (2010). Melt-Extruded Solid Dispersions Containing an Apoptosis-Inducing Agent. U.S. Patent.

[39] Breitenbach J., Alani L., Berndl G., Gosh S., Liepold B., Reinhold U., Rosenberg J. (2003). Abbott Laboratories assignee. Solid dosage pharmaceutical form. U.S. Patent.

[40] Harmon P.A., Variankaval N. (2013). Solid Dosage Formulations of an Orexin Receptor Antagonist. U.S. Patent.

[41] Rosenberg J., Berndl G., Neumann J., Breitenbach J. (2002). Compositions and Dosage Forms for Application in the Oral Cavity in the Treatment of Mykoses. WIPO Patent.

[42] Sever N., Westedt U., Lander U., Schneider K., Steitz B., Mueller T., Reul R., Obermiller C., Jayasankar A., Simon M. (2016). Solid Pharmaceutical Compositions for Treating HCV. WIPO Patent.

[43] Lu J., Obara S., Ioannidis N., Suwardie J., Gogos C., Kikuchi S. (2018). Understanding the Processing Window of Hypromellose Acetate Succinate for Hot-Melt Extrusion, Part I: Polymer Characterization and Hot-Melt Extrusion. Adv. Polym. Technol..

