# Supplementary material for: A Hot-Melt Extrusion Risk Assessment Classification System for Amorphous Solid Dispersion Formulation Development
Source: Pharmaceutics. 2022 May 12;14(5):1044. doi: 10.3390/pharmaceutics14051044 (PMC9147278; doi:10.3390/pharmaceutics14051044)
Supplement: Supplementary file 1 [file pharmaceutics-14-01044-s001.zip › pharmaceutics-1712420-supplementary.pdf]

Supporting Information for:

A Hot-Melt Extrusion Risk Assessment Classification System for Amorphous Solid Dispersion Formulation Development

Samuel O. Kyeremateng, Kristin Voges, Stefanie Dohrn, Ekaterina Sobich, Ute Lander, Stefan Weber, David Gessner, Rachel C. Evans, Matthias Degenhardt*

AbbVie Deutschland GmbH & Co. KG, Global Pharmaceutical R&D, Knollstraße, D-67061 Ludwigshafen am Rhein, Germany.

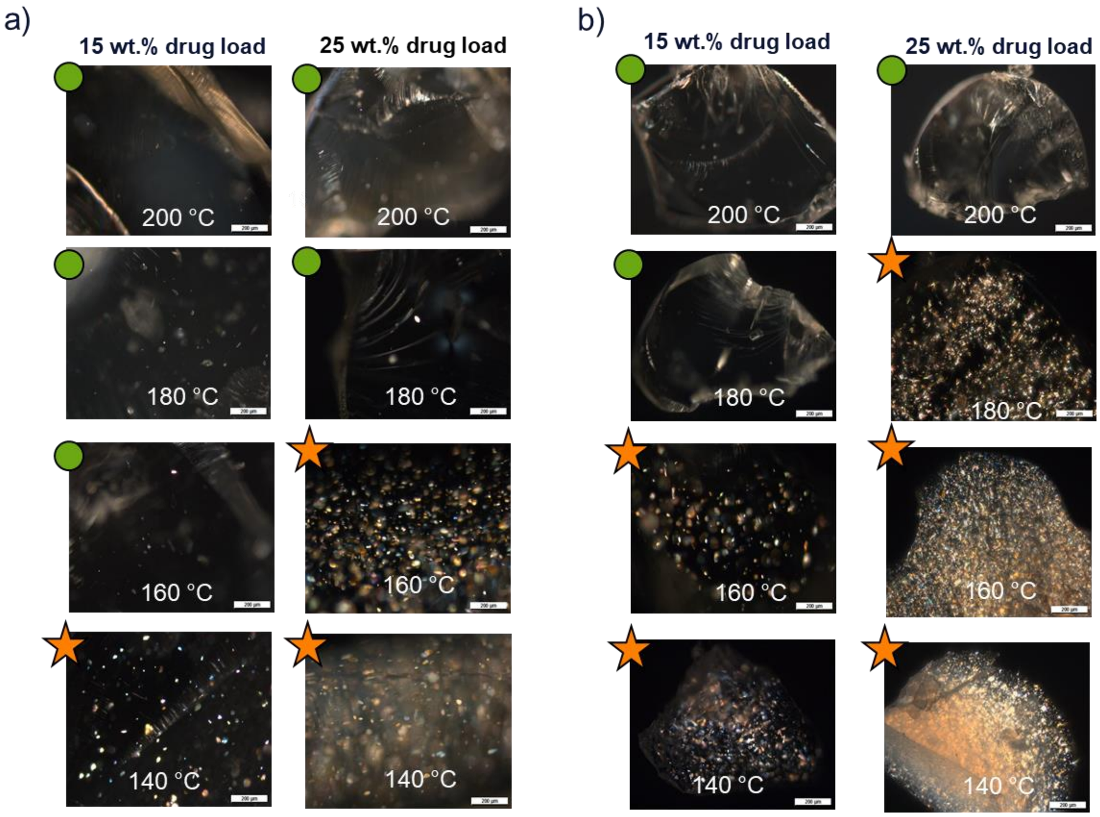

Figure S1: Polarized light microscopy (PLM) images of extrudates; 15 wt.% and 25 wt. % sulfamerazine (**a**) in Copovidone and (**b**) in Soluplus. The green circle and orange star symbols reflect PLM amorphous and crystalline extrudates, respectively, after HME at 180 °C, 200 °C, 220 °C, and 230 °C.

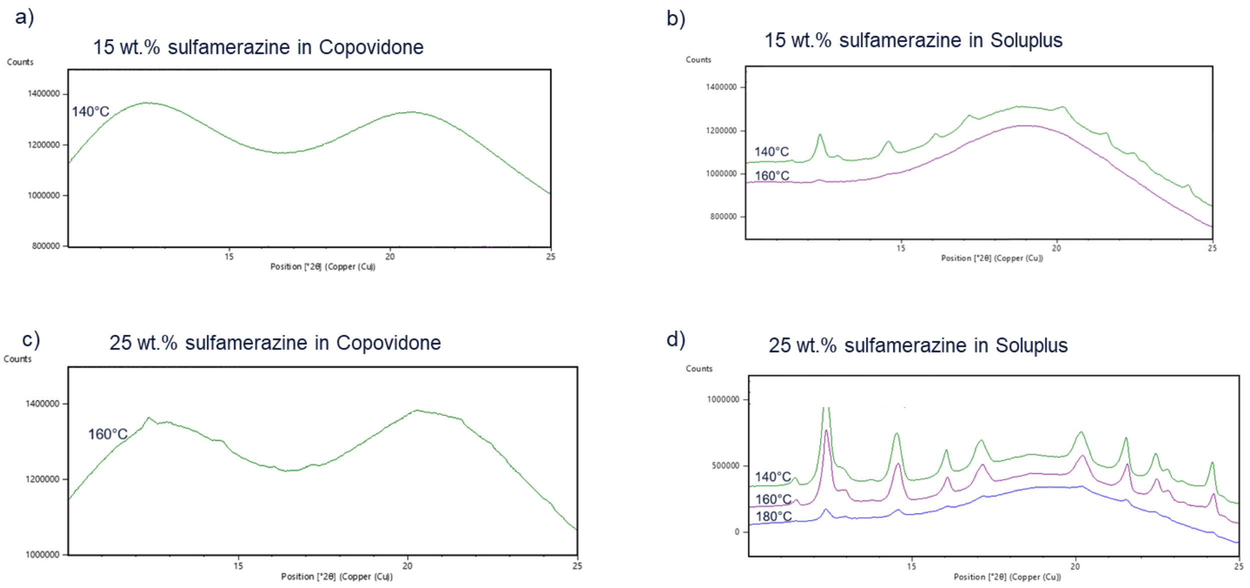

Figure S2: XRPD data of extrudates; 15 wt.% sulfamerazine (**a**) in Copovidone and (**b**) in Soluplus and 25 wt.% sulfamerazine in (**c**) Copovidone and (**d**) Soluplus.

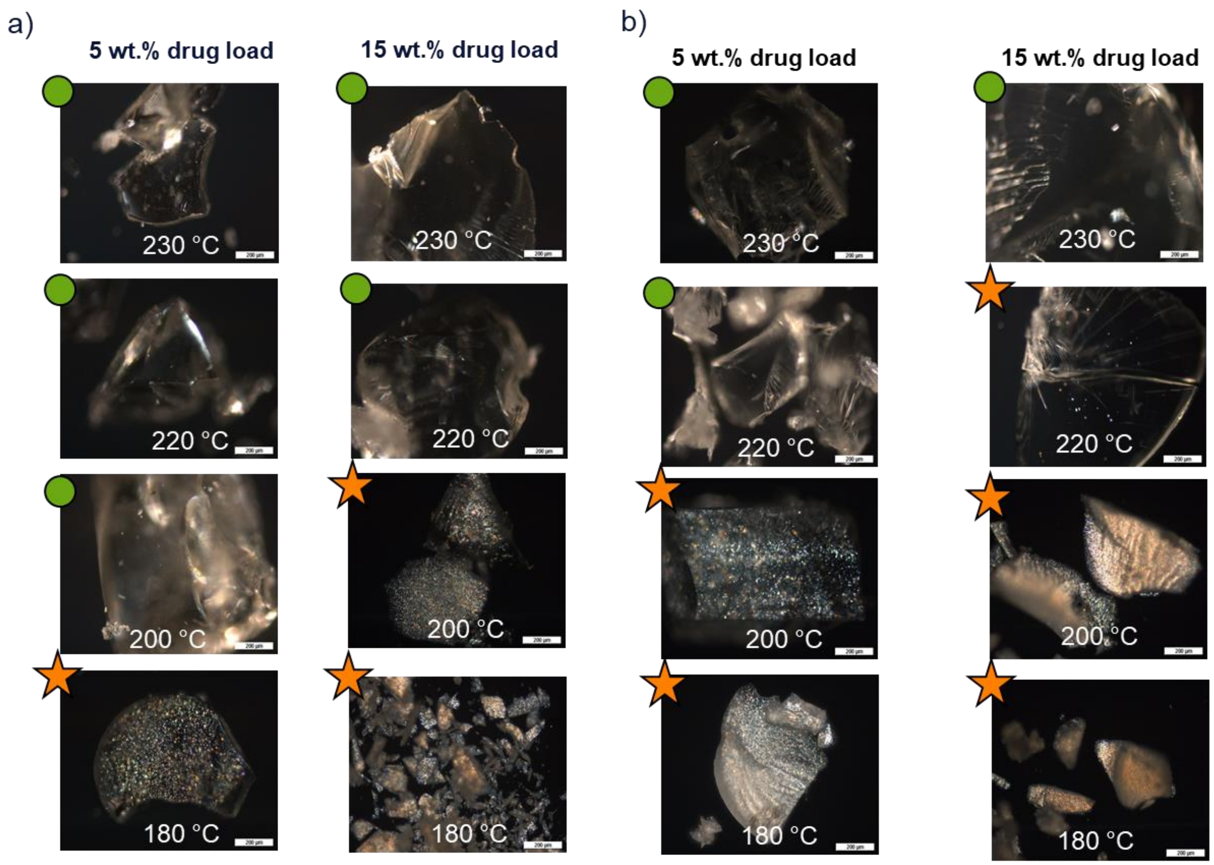

Figure S3: Polarized light microscopy (PLM) images of extrudates; 5 wt.% and 15 wt. % telmisartan (**a**) in Copovidone and (**b**) in Soluplus. The green circle and orange star symbols reflect PLM amorphous and crystalline extrudates, respectively, after HME at 180 °C, 200 °C, 220 °C, and 230 °C.

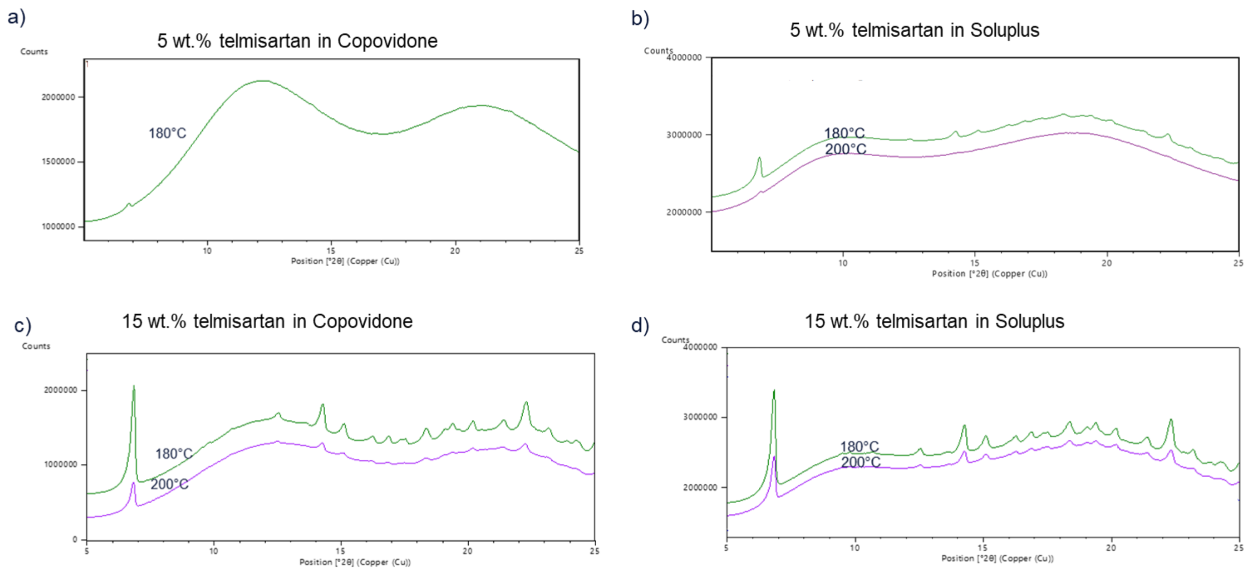

Figure S4: XRPD data of extrudates; 5 wt.% telmisartan (**a**) in Copovidone and (**b**) in Soluplus and 15 wt.% telmisartan in (**c**) Copovidone and (**d**) Soluplus.
